# FRET-Based Sorting of Live Cells Reveals Shifted Balance between PLK1 and CDK1 Activities During Checkpoint Recovery

**DOI:** 10.3390/cells9092126

**Published:** 2020-09-19

**Authors:** Lorenzo Lafranchi, Erik Müllers, Dorothea Rutishauser, Arne Lindqvist

**Affiliations:** 1 Department of Cell and Molecular Biology, Karolinska Institutet, SE-171 77 Stockholm, Sweden; lorenzo.lafranchi@scilifelab.se (L.L.); erik.muellers@astrazeneca.com (E.M.); 2 Division of Physiological Chemistry I, Department of Medical Biochemistry and Biophysics, Karolinska Institutet, SE-171 77 Stockholm, Sweden; dorothea.rutishauser@gmail.com; 3 Science for Life Laboratory, SE-171 65 Stockholm, Sweden

**Keywords:** G2, mitotic entry, checkpoint recovery, FRET, PLK1, Cyclin B1, CDK1, DNA damage, mitosis

## Abstract

Cells recovering from the G2/M DNA damage checkpoint rely more on Aurora A-PLK1 signaling than cells progressing through an unperturbed G2 phase, but the reason for this discrepancy is not known. Here, we devised a method based on a FRET reporter for PLK1 activity to sort cells in distinct populations within G2 phase. We employed mass spectroscopy to characterize changes in protein levels through an unperturbed G2 phase and validated that ATAD2 levels decrease in a proteasome-dependent manner. Comparing unperturbed cells with cells recovering from DNA damage, we note that at similar PLK1 activities, recovering cells contain higher levels of Cyclin B1 and increased phosphorylation of CDK1 targets. The increased Cyclin B1 levels are due to continuous Cyclin B1 production during a DNA damage response and are sustained until mitosis. Whereas partial inhibition of PLK1 suppresses mitotic entry more efficiently when cells recover from a checkpoint, partial inhibition of CDK1 suppresses mitotic entry more efficiently in unperturbed cells. Our findings provide a resource for proteome changes during G2 phase, show that the mitotic entry network is rewired during a DNA damage response, and suggest that the bottleneck for mitotic entry shifts from CDK1 to PLK1 after DNA damage.

## 1. Introduction

The levels and activities of many proteins change through the cell cycle. These variations are needed to direct events such as DNA replication and cell division in a timely fashion. Not surprisingly, changes in protein levels and activities are under strict control. After an initial external regulation by growth factors, the changes are intrinsically enforced [1]. In this sense, the cell cycle constitutes a continuous oscillation of protein levels and activities. A complex mix of feedback and feedforward systems ensures that protein levels and activities change until being reset after cell division [2,3]. In G2 phase, feedback loops between Cyclin–Cyclin Dependent Kinases (CDK) and transcription factors such as Forkhead Box Protein M1 (FoxM1) ensure a continuous increase in protein levels required for mitosis [4,5,6]. The initiation of these loops is regulated by a feedforward system that functions as a brake while DNA replication is ongoing [3,7,8]. However, a detailed understanding of the mechanisms that steer changes through G2 phase is lacking, primarily due to a lack of time resolved data on changes in protein levels and activities that occur within cell cycle phases.

The oscillation of protein levels and activities caused by feedback and feedforward systems constitutes a challenge when the cell cycle needs to be paused; it is not easy to freeze a complex free-running self-sustained oscillator [9]. Even if the oscillation is stopped, the system is likely to keep changing towards a new steady state. In this sense, various cell synchronization methods have been criticized for creating an artificial situation [10]. Indeed, recent evidence indicates that even a pause before initiating the cell cycle (i.e., entry to quiescence) will affect how the following S-phase is performed [11]. A prime example of a cell cycle pause is a DNA damage checkpoint. After DNA damage in G2 phase, a variety of mechanisms cooperate to inhibit activities that enforce mitosis [12]. Interestingly, when cells recover from the DNA damage checkpoint and resume the cell cycle, the requirements for individual proteins seem to have changed. In particular, the kinases Polo Like Kinase 1 (PLK1) and Aurora kinase A, and the phosphatase Cell Division Cycle 25B (CDC25B) have been reported to be more important for mitotic entry after recovery from a checkpoint than during an unperturbed G2 phase [13,14]. The reason for this discrepancy remains unclear, although the identification of a role for PLK1 in reversing checkpoint signaling points to specific functions related to the DNA damage response [15,16,17,18]. However, whether the cell cycle pause caused by activation of a DNA damage checkpoint leads to changed protein balances during checkpoint recovery remains unclear.

We have devised a new method to sort cells based on Förster Resonance Energy Transfer (FRET). We used a FRET-based reporter for PLK1 activity to separate fractions of live unsynchronized cells at different stages within G2 phase. Employing mass spectrometry, we identified changes in protein levels that occur during G2 phase. Further, we compared unperturbed cells and cells recovering from a DNA damage checkpoint that, based on the PLK1 reporter, were in similar positions within G2 phase. We noted that cells recovering from a DNA damage checkpoint contained higher levels of Cyclin B1. The increased Cyclin B1 levels were due to continuous production during the DNA damage dependent checkpoint. Cells entering mitosis after recovery from checkpoint activation contained higher Cyclin B1 levels than unperturbed mitotic cells, showing that the DNA damage response in G2 phase affects protein composition in the following mitosis. In addition to a change in protein levels, we found that the balance between PLK1 target phosphorylation and Cyclin-CDK target phosphorylation is altered after recovery from the DNA damage checkpoint in G2 phase. In line with this changed balance, we find that not only are cells recovering from DNA damage more sensitive to a PLK1 inhibitor, they are also less sensitive to inhibition of CDK activity.

## 2. Materials and Methods

### 2.1. Cell Culture

U2OS cell lines were maintained in Dulbecco’s Modified Eagle Medium (DMEM), containing high glucose, GlutaMAX^TM^ and pyruvate (Gibco, Waltham, MA, USA). The medium was supplemented with 6% heat-inactivated fetal bovine serum (FBS, HyClone, Logan, UT, USA) and 1% Penicillin/Streptomycin (Pen/Strep, HyClone). RPE cell lines were maintained in DMEM/Nutrient mixture F-12, containing GlutaMAX^TM^ (Gibco). Medium was supplemented with 10% FBS and 1% Pen/Strep. All cell lines were cultured in an ambient-controlled incubator at 37 °C and 5% CO_2_.

### 2.2. Flow Cytometry

Cells were harvested with trypsin/EDTA (Gibco) at the indicated time points after treatment and fixed in ice-cold 70% ethanol (Sigma-Aldrich, St. Louis, MO, USA). After fixation, cells were stained with rabbit Histone H3 pS10, subsequently stained with Alexa647- conjugated secondary antibodies (Invitrogen, Waltham, MA, USA), and counterstained with propidium iodide (Sigma-Aldrich). Cells were analyzed on a FACSCanto II (Becton Dickinson, Franklin Lakes, NJ, USA). Per sample, a minimum of 10^4^ events was analyzed.

### 2.3. FRET-Activated Cells Sorting

Cells were harvested with trypsin/EDTA and re-suspended in PBS (Gibco). Live cells were sorted in PBS using a FACSAria III sorter (Becton Dickinson) using a 100 mm nozzle at 20 psi. Cells expressing the PLK1 probe at a low level were gated out from the analysis to decrease the noise in the readout. FRET ratio was recorded using F47-470 and F47-535 filters (AHF Analysentechnik, Tübingen, Germany). Sorting periods were limited to a maximum of 20 min per sample. Right after, cells were spun down and snap frozen in liquid nitrogen. For immunofluorescence analysis, cells were processed as described for quantitative immunofluorescence.

### 2.4. Protein Digestion for Mass Spectrometry Analysis

Cell pellets of about 200,000 cells per sample in 30 μL were thawed on ice and reconstituted in 90 μL 8M Urea with 150 mM NaCl, resulting in 120 μL of 6M Urea. The cells were disrupted by probe sonication (Vibra-Cell™ CV18, Sonics & Materials, Newtown, CT, USA), and cell debris was removed by short centrifugation at 4 °C. In total, 5 micrograms of extracted protein from each sample were dissolved in 0.1% ProteaseMAX™ (Promega Co., Madison, WI, United States), 50 mM ammonium bicarbonate and 10% acetonitrile. The resulting protein solutions were incubated for 30 min at 50 °C while shaking, followed by an additional bath sonication of 10 min at room temperature. Samples were centrifuged and the supernatant was directly subjected to a tryptic digestion protocol carried out by a liquid handling robot (MultiProbe II, Perkin Elmer, Waltham, MA, USA). This included protein reduction in 5 mM DTT at 56 °C for 30 min and alkylation in 15 mM iodoacetamide for 30 min at room temperature in the dark. Trypsin was added in an enzyme-to-protein ratio of 1:30, and digestion was carried out over night at 37 °C.

### 2.5. Liquid Chromatography Tandem Mass Spectrometry

Tryptic peptides were acidified, centrifuged and cleaned with a HyperSep™ C18 Plates (Thermo Fisher Scientific, Waltham, MA, USA). About 1 μg of the resulting peptide mixture was injected into a nano-Ultimate HPLC system (Thermo Fisher Scientific) in-line coupled to a QExactive mass spectrometer (Thermo Fisher Scientific). The chromatographic separation of the peptides was achieved using a 28 cm long in-house packed column (C18-AQ ReproSil-Pur^®^, Dr. Maisch GmbH, Ammerbuch, Germany) with the following gradient: 7−28% acetonitrile (ACN) in 115 min and 28−96% ACN in 5 min at a flow rate of 300 nL/min.

The MS acquisition method was comprised of one survey full scan ranging from *m*/*z* 300 to *m*/*z* 1650 acquired with a resolution of R = 140,000 at *m*/*z* 400, followed by data-dependent HCD scans from a maximum of sixteen of the most intense precursor ions with a charge state ≥2. MS2 scans were acquired with a resolution of R = 17,500, a target value of 2e5, isolation width was set to 4, and normalized collision energy to 26.

### 2.6. Mass Spectrometry Data Analysis

Tandem mass spectra were extracted using Raw2MGF [19] and the resulting mascot generic files were searched against a SwissProt protein database using the Mascot 2.5.1 (Matrix Science Ltd., London, UK). Mascot was set up to search a concatenated SwissProt protein database (selected for Homo sapiens) using trypsin and allowing for two missed cleavage sites. Peptide mass tolerance was set to 10 ppm, and fragment ion mass tolerance to 0.02 Da. Carbamidomethylation of cysteine was specified as a fixed modification, whereas oxidation of methionine and deamidation of asparagine and glutamine were defined as variable modifications.

Quantitative information was extracted using Quanti 2.5.4 [19]. This software performs extracted ion current quantification for label-free quantitation, and only peptides identified with a Mascot score higher than 13.6 were selected. Such a threshold was set to fulfill the condition of no more than 1% FDR over the total peptide population. Only proteins quantified with at least two such peptides were considered for quantitation. Normalization of the data was done by calculating the summed intensities of all proteins in each sample and the median of all these summed intensities over the entire sample set. Each quantitative value was multiplied by the median/summed intensity and the resulting values were log10 transformed.

The correlation factor (cf) utilizes the coefficient of determination (r^2^) of a simple linear regression model. The r^2^ value indicates the proportion of the variance in the dependent variable (protein levels) that is predictable from the independent variable (progressive G2 fractions). We calculated cf equal to r^2^ for positive correlations (increasing protein levels) and equal to −r^2^ for negative correlations (decreasing protein levels).

Full results of the mass spectrometry analysis are available as supplementary data file.

### 2.7. Live Cell Microscopy and Quantitative Immunofluorescence (qIF)

Live cell imaging was performed on a DMI6000 Imaging System (Leica, Wetzlar, Germany) using a 20×, NA 0.40 objective. Cells were constantly kept in a humidified, 37 °C chamber at 5% CO_2_. To quantify the cumulative mitotic entry, cells were plated ∼24 h prior to imaging in full-growth media in a 96-well plate (BD Falcon, Corning Inc., Corning, NY, USA) such that the density would remain sub-confluent until the end of the imaging period.

For immunofluorescence, cells grown in a 96-well plate (BD Falcon) were fixed in 3.7% formaldehyde (Sigma-Aldrich) for 5 min at room temperature and permeabilized for 2 min with ice-cold methanol. For experiments using EdU staining, EdU (5-ethynyl-2′-deoxyuridine, Invitrogen Molecular Probes, Carlsbad, CA, United States) was added one hour before fixation. Prior to incubation with the appropriate antibodies, cells were blocked for 1 h in 2% BSA (Sigma-Aldrich) in TBS supplemented with 0.1% Tween-20 (TBS-T). Cells were incubated with primary antibodies o.N. at 4 °C. After washing with TBS-T, cells were stained with Alexa488-, Alexa568-, or Alexa647-conjugated secondary antibodies (Invitrogen) for 60 min at room temperature and counterstained with 0.5 ug/mL DAPI (Sigma-Aldrich). After washing with TBS-T, images were obtained using a ImageXpress system (Molecular Devices, San Jose, CA, United States) using a 20×, NA 0.45 objective. For quantifying 53BP1 foci, cells were grown on glass coverslips. After fixation and staining as described above, coverslips were mounted and sealed with Vectashield (Vector Laboratories, Burlingame, CA, United States) containing DAPI. Images were acquired on a DeltaVision Spectris imaging system (GE healthcare, Chicago, IL, USA) using a 40× oil immersion, NA 1.35 objective. Image analysis was performed using ImageJ [20] and Cell Profiler [21]. Estimation of time-course from fixed cells was performed as in [22], using DAPI and Cyclin B1 stainings to order cells.

### 2.8. Chemicals and Inhibitors

Where indicated, cells were treated with Neocarzinostatin (NCS, Sigma-Aldrich), *S*-Trityl-L-cysteine (STLC, Tocris bioscience, Bristol, UK), BI 2536 (Selleckchem, Houston, TX, USA), RO-3306 (Calbiochem, Burlington, MA, USA), MG-132 (Sigma-Aldrich), cycloheximide (Sigma-Aldrich)), and DMSO (Sigma-Aldrich)).

### 2.9. Western Blotting (WB)

Cell extracts were prepared in Laemmli buffer (4% SDS, 20% glycerol, 120 mM Tris–HCl pH 6.8) supplemented with Pierce^TM^ reducing sample buffer (Thermo Scientific, Waltham, MA, USA). Proteins were resolved by SDS–PAGE using precasted polyacrylamide gels (Biorad, Hercules, CA, USA) and transferred to PVDF membranes (Biorad). Immunoblots were performed using the appropriate primary antibodies and the relative HPR-coupled secondary antibodies (GE Healthcare, Chicago, IL, USA). Proteins were visualized on a ChemiDoc MP imaging system (Biorad) using the ClarityTM Western ECL Blotting Substrate (Biorad). Antibodies are specified in Table 1.

## 3. Results

### 3.1. A Setup to Sort Cells Based on FRET

**Figure 1:**
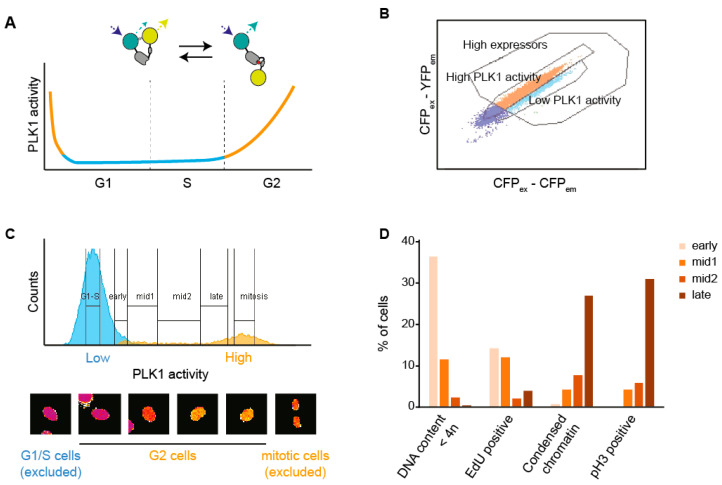
FRET-activated cell sorting (FRACS) is used to collect cells with different levels of PLK1 activity. (**A**) Schema of the FRET probe used throughout the experiments. The probe contains a consensus motif for PLK1 in the linker region between the two FRET fluorophores. The conformational change induced by phosphorylation results in a decreased FRET ratio. The graph represents the levels of PLK1 activity that are associated with the various phases of the cell cycle. (**B**) Flow cytometry was used to visualize an asynchronous population of RPE1 cells expressing the PLK1 FRET probe depicted in (**A**). Cells expressing the probe at high level were selected to ensure a robust readout. (**C**) Inverted FRET ratio (PLK1 activity) of an asynchronous population of cells measured by flow cytometry. Based on the knowledge that PLK1 activity increases during G2 phase, we were able to discriminate and collect fractions of G1-S (no/low PLK1 activity, cyan peak), early-G2 (low PLK1 activity), mid-G2 (medium PLK1 activity) and late-G2 (high PLK1 activity) cells. Representative pictures of cells belonging to each fraction are shown below the histogram. False coloring was used to render the changes in PLK1 activity (purple = low, yellow = high). (**D**) Asynchronously-growing RPE1 cells were pulsed 1 h with EdU prior to be sorted based on their PLK1 activity level. Right after FACS, the different populations of cells were fixed and prepared for microscopy analysis. At least 50 cells were quantified to assess their DNA content, the degree of DNA condensation, phosphorylation of Histone H3 and the presence of EdU. Representative pictures are shown in Figure S1D.

We developed a setup to use Fluorescence Activated Cell Sorting (FACS) of live cells based on Förster Resonance Energy Transfer (FRET). We optimized the setup for cells expressing a FRET-based biosensor for PLK1 target phosphorylation. The PLK1 biosensor show a low inverted FRET-ratio through most of G1 and S-phase, and a gradual increase from the S/G2 transition until mitosis [14,23] (Figure 1A). For simplicity, we refer to inverted FRET-ratio as PLK1 activity. By applying ratiometric flow cytometry, we found that both U2OS and RPE1 cells, expressing either a diffusible or a Histone H2B-coupled PLK1 biosensor, showed a similar pattern of PLK1 activity (Figure 1B,C and Figure S1A). As expected, measured PLK1 activity was sensitive to the addition of a PLK1 inhibitor (Figure S1B). A majority of cells show low PLK1 activity, consistent with a majority of cells being in G1 and S phases (Figure 1C and Figure S1A). A small population showed high PLK1 activity, and this population increased after blocking cells in mitosis with S-trityl-L-cysteine (STLC), consistent with the stable high PLK1 activity detected in mitotic cells (Figure 1C and Figure S1A,B). In between these peaks there is a distribution of cells, consistent with cells progressing through G2 phase or exiting mitosis. The pattern of PLK1 activity stayed stable throughout the cell sorting (Figure S1C). We conclude that it is possible to use ratiometric cytometry to detect PLK1 activity in living cells.

We reasoned that PLK1 activity could be used to separate cells at different stages within G2 phase, therefore we sorted RPE1 PLK1-FRET cells into four populations with intermediate/increasing PLK1 activity (Figure 1C). A majority of cells in the four populations showed 4N DNA content, suggesting that the contribution of cells reversing PLK1 activity after mitotic exit is limited. Further, a majority of cells did not contain EdU staining, condensed chromatin or phospho-Histone H3 staining, indicating that the populations are enriched for G2 cells (Figure 1D and Figure S1D). The populations with lowest PLK1 activity showed a higher presence of EdU positive cells and cells with less than 4N DNA content, likely reflecting that they are adjacent to the G1/S population. Similarly, the populations with highest PLK1 activity showed a higher presence of condensed chromatin and phospho-Histone H3 staining, likely reflecting that they are adjacent to the population of mitotic cells (Figure 1D and Figure S1D). We conclude that FACS based on PLK1-FRET allows enrichment of G2 cells and separation of cells within G2 phase.

### 3.2. Characterization of Changes in Protein Content Through G2 Phase

**Figure 2:**
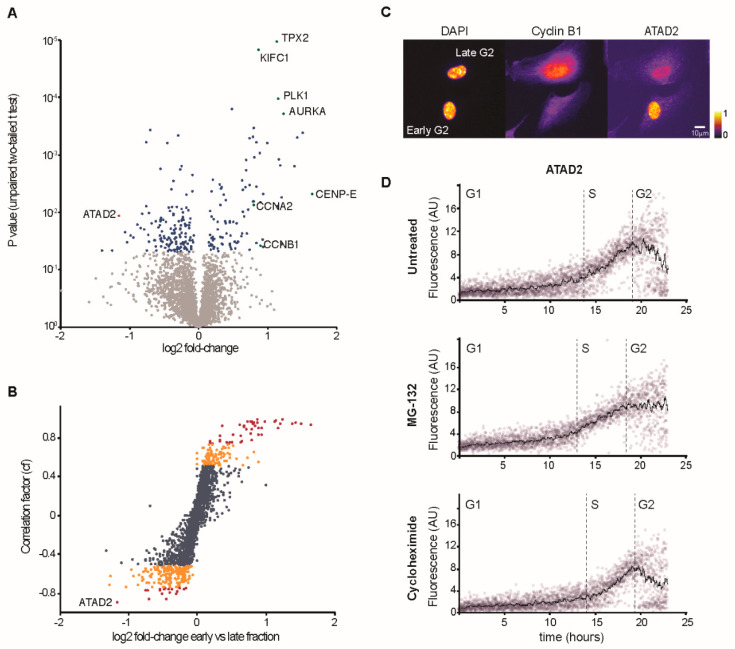
Quantitative analysis of G2 progression. (**A**) Volcano plot illustrating significantly differentially expressed proteins between early and late G2 phase. The −log10 corrected P value is plotted against the log2 fold change. Blue coloring denotes P = 0.05, which is our significance threshold (prior to logarithmic transformation). (**B**) Protein changes through G2 phase. Correlation factor (cf) indicating steady linear change in protein expression over the four G2 fractions plotted against the log2 fold change. Orange coloring denotes robust linear correlation (cf > 0.5 or cf < −0.5). Red coloring denotes strong linear correlation (cf > 0.75 or cf < −0.75). (**C**) Representative images of asynchronously growing RPE1 cells. Cells were fixed and stained with antibodies against Cyclin B1 and ATAD2. Cells in early G2, characterized by low levels of Cyclin B1 staining, display high levels of ATAD2. Cells in late G2, characterized by high levels of Cyclin B1, display low levels of ATAD2. (**D**) Quantitative immunofluorescence of ATAD2 levels throughout the cell cycle. Two hours prior to fixation asynchronously growing RPE1 cells were treated with either DMSO, 10 µM MG-132 or 10 g/mL cycloheximide (CHX). Samples were stained with antibodies against Cyclin B1 and ATAD2, and counterstained with DAPI. Following image acquisition and quantification, cells were sorted in silico and assigned to their position within the cell cycle based on their DAPI and cyclin B1 intensity levels (Figure S2B). Every dot on the plot represents a single cell and the trend line is the running average of 30 cells.

We next sought to assess protein changes that occur through G2 phase. We sorted RPE1 H2B-PLK1-FRET cells into the four populations that were enriched for different stages of G2 cells (Figure 1C) and analyzed protein content using mass spectroscopy. We calculated a correlation factor (cf) that indicates whether proteins show a steady linear change through the four G2 phase populations. In all samples combined, we identified 2493 proteins based on at least two unique peptides. Whereas a majority of proteins did not change between the four samples, we found that known key G2 regulators as Cyclin A2, Cyclin B1, PLK1, and Aurora A increased successively as PLK1 activity increased (Figure 2A and supplementary data file). We note that a majority of the proteins that exhibited large changes between the first and the last population also showed a large correlation factor (Figure 2B). This shows that the four samples are enriched in distinct populations of G2 cells.

To test the predictive power of our findings, we focused on proteins decreasing through G2 phase, as they are less well-characterized compared to proteins that increase during G2 phase. We selected ATAD2, which showed a large negative correlation factor and fold change (Figure 2B and Figure S2A). Using immunofluorescence, we note that ATAD2 staining decreases as Cyclin B1 staining is increasing, indicating that ATAD2 levels are lower in late G2 phase compared to in early G2 phase (Figure 2C). To characterize ATAD2 dynamics, we quantified the immunofluorescence signal and created a time-course based on the increasing presence of Cyclin B1 through the cell cycle (Figure 2D and Figure S2B). We found that ATAD2 levels were low in G1 phase, increased throughout S-phase, and decreased in G2 phase (Figure 2D). The reduction in ATAD2 through G2 phase was prevented by the addition of the proteasome inhibitor MG-132 but was unaffected by the addition of the translation inhibitor Cycloheximide, suggesting that ATAD2 levels decrease through G2 phase due to protein degradation. We conclude that the setup allows sorting of distinct populations within G2 phase, and that the mass spectrometry data can function as a reference for protein changes within G2 phase (supplementary data file).

### 3.3. Increased Cyclin B1 Levels During DNA Damage Checkpoint Recovery

Spontaneous recovery from a DNA damage checkpoint in G2 phase is characterized by a gradual increase in PLK1 activity. The increase is slow, can last up to 15 h, and is highly asynchronous, which represents an obstacle for analysis [24,25]. We reasoned that our setup could be used to enrich and collect cells at specific stages of spontaneous checkpoint recovery. We titrated the radiomimetic drug Neocarzinostatin (NCS) in U2OS PLK1-FRET cells to define conditions for which a cell cycle arrest is robustly enforced and spontaneous recovery occurs. We note that after treatment with low dose of NCS (1–2 nM), cells delayed cell cycle progression, and after approximately 6 h resumed cell division (Figure S3A). NCS addition caused a temporal increase in 53BP1 foci, indicating that DNA breaks were induced and subsequently repaired (Figure S3B). Using ratiometric flow cytometry, we detected a reduction of cells with high PLK1 activity 4 h after addition of NCS, indicating that a DNA damage checkpoint was initiated (Figure S3C). A total of ten hours after addition of NCS, high PLK1 activity resumed in a dose-dependent manner, indicating that a subset of cells was recovering from a DNA damage checkpoint. We conclude that it is possible to follow DNA damage checkpoint recovery in G2 phase by ratiometric flow cytometry.

**Figure 3:**
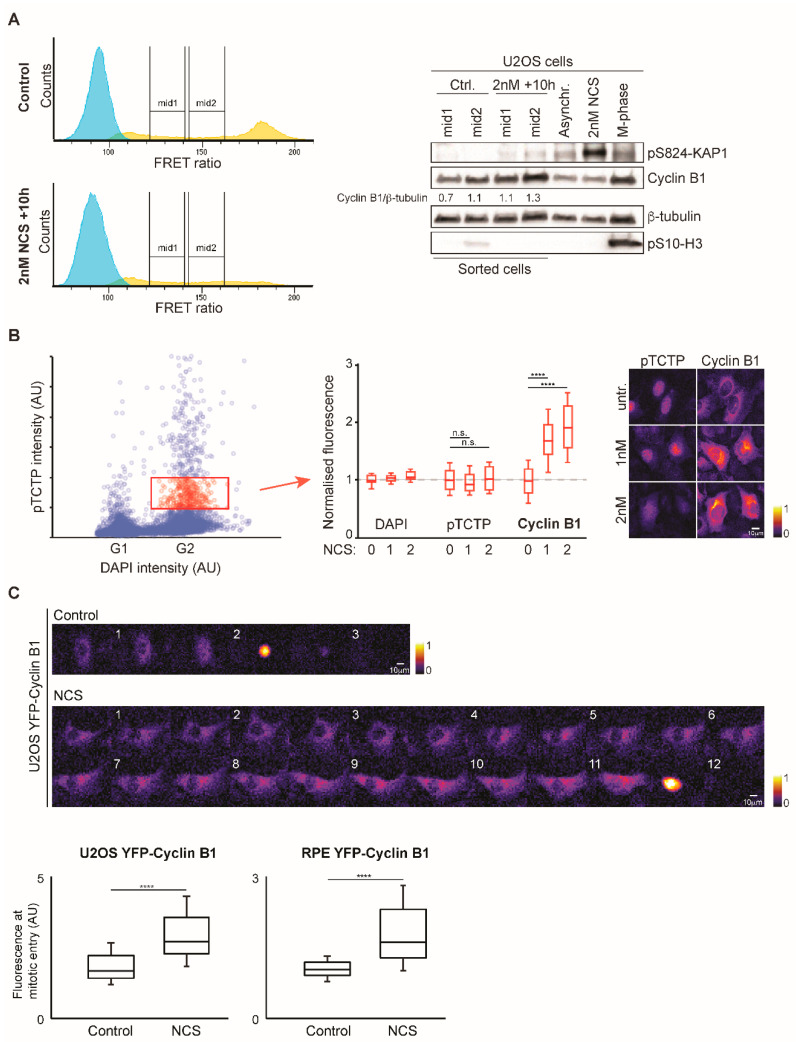
Cells recovering from a DNA damage checkpoint contain increased level of Cyclin B1. (**A**) 10 h after being exposed to 2 nM NCS, cells were sorted according to their level of PLK1 activity. Two fractions, defined “mid1” and “mid2”, were collected for both control and NCS-treated cells. All samples were treated with 10 µM S-trityl-L-cysteine (STLC) 4 h before the sorting. After the sorting, cells were pelleted, lysed in Laemmli sample buffer and analyzed by Western blot. Unsorted cells, either untreated, treated with 2 nM NCS or treated with 10 µM STLC and subjected to mitotic shake off were included as a reference. Phosphorylation of histone H3 was used as a marker of mitotic cells, whereas phosphorylation of KAP1 (a direct target of the ataxia-telangiectasia mutated (ATM) kinase) was used to indicate activation of the DNA damage response. (**B**) 10 h after treatment with 2 nM NCS, U2OS cells were fixed and stained with phosphorylated TCTP (pTCTP, a proxy for PLK1 activity) and Cyclin B1 antibodies. Integrated intensities of DAPI and pTCTP were used to identify early G2 cells (red dots, enclosed by the red rectangle), whose levels of DAPI, pTCTP and cyclin B1 are plotted in the graph on the right. Values are normalized to the average intensity in the absence of NCS. Representative images are showed to the right. (**C**) U2OS and RPE1 cells carrying an endogenously YFP-tagged Cyclin B1 were treated with 1 nM NCS and followed during the recovery phase by live cell microscopy. Graphs present the means of the integrated intensity of nuclear Cyclin B1-YFP. 34 to 92 cells were quantified for each condition and error bars represent the standard deviation. Representative images of U2OS cells are shown on top of the panel. Timestamps on the top-left corner indicate hours post damage induction. n.s. (not significant) indicates *p* > 0.05, whereas **** indicates *p* < 0.0001 using Student’s *T*-test.

To study the difference between unperturbed cells and cells recovering from a DNA damage checkpoint, we sorted cells 10 h after NCS addition. We collected cells at two ranges of intermediate PLK1 activity, corresponding to earlier (“mid1”) and later (“mid2”) stages of G2 phase. Analyzing the protein content by Western blot, we noticed that although based on populations with similar PLK1 activity, the recovering populations contain increased levels of Cyclin B1 (Figure 3A). To independently test if recovering cells at similar PLK1 activities contain different levels of Cyclin B1, we subjected NCS-treated cells to quantitative immunofluorescence. Using levels of phosphorylated Translationally-Controlled Tumor Protein Homolog (TCTP) as proxy for PLK1 activity [26], we find that for similar levels of phosphorylated TCTP, the Cyclin B1 levels were higher in recovering cells than in unperturbed cells (Figure 3B). As yet an independent test, we quantified Cyclin B1-YFP levels in U2OS and RPE cells, created by gene targeting in the endogenous locus [23]. In both cell types, Cyclin B1-YFP levels were higher in mitotic cells that had recovered from NCS addition (Figure 3C). Following the cells over time using live cell imaging, we noted that the difference was due to a continuous production of Cyclin B1-YFP during the prolonged G2 phase caused by NCS addition (Figure 3C). Thus, cells that recover from DNA damage show a different balance between PLK1 activity and Cyclin B1 levels compared to unperturbed cells.

### 3.4. The Balance Between CDK1 and PLK1 is Altered During DNA Damage Checkpoint Recovery

**Figure 4:**
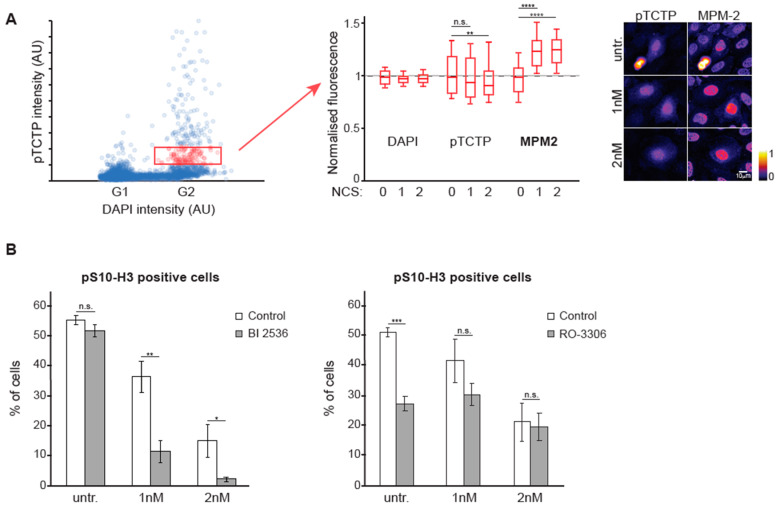
The balance between PLK1 and CDK1-mediated phosphorylation is altered during recovery. (**A**) 10 h after treatment with 2 nM NCS, U2OS cells were fixed and stained with phosphorylated TCTP (pTCTP, a proxy for PLK1 activity) and MPM-2 (a proxy for CDK1 activity) antibodies. Integrated intensities of DAPI and pTCTP were used to identify early G2 cells (indicated in red rectangle), whose levels of DAPI, pTCTP and MPM-2 are plotted in the graph on the right. Values are normalized to the average intensity in the absence of NCS. Representative images are shown to the right. (**B**) U2OS cells were treated with different concentrations of NCS, and after 4 h were either cotreated with DMSO, 10 nM of the PLK1 inhibitor BI 2536 (left panel) or 5 µM of the CDK1 inhibitor RO-3306 (right panel). 16 h after damage induction, cells were fixed and stained with a pS10-histone H3 specific antibody. All samples were treated with 10 µM of S-trityl-L-cysteine (STLC) to trap cells in mitosis. Samples were analyzed by flow cytometry. The graph shows the mean and standard error of 3 independent experiments. n.s. (not significant) indicates *p* > 0.05, * indicates p < 0.05, ** indicates *p* < 0.01, *** indicates *p* < 0.001 and **** indicates *p* < 0.0001 using Student’s *T*-test.

Accumulating kinase activity of CDK1 and PLK1 are key drivers of mitotic entry. Having observed higher levels of Cyclin B1 in mitotic entry after recovery from a DNA damage checkpoint compared to unperturbed mitotic entry, we wanted to test if the balance between CDK1 activity and PLK1 activity was altered during recovery from a DNA damage checkpoint. We used quantitative immunofluorescence of phosphorylated TCTP and MPM2, a proxy for CDK1 activity [27]. In unperturbed cells phospho-TCTP levels increase through G2 phase [7] and MPM2 levels increased concomitantly in a nonlinear manner (Figure S4A). Interestingly, cells recovering from a DNA damage checkpoint showed a different balance between phospho-TCTP and MPM2: at similar phospho-TCTP levels, the MPM2 levels were higher in recovering cells compared to unperturbed cells (Figure 4A and Figure S4A). This indicates that CDK1 activity is higher relative to PLK1 activity after recovery from a DNA damage checkpoint, compared to during an unperturbed G2 phase.

To test whether the changed balance in kinase activities is translated to changed sensitivity toward kinase inhibitors, we used small-molecule inhibitors to reduce PLK1 and CDK1 activity. As previously shown, we found that cells are more sensitive to PLK1 inhibition after recovery from a DNA damage checkpoint than during unperturbed mitotic entry [13] (Figure 4B, left panel). Correspondingly, we find that cells are less sensitive to inhibition of CDK1 activity after recovery from a DNA damage checkpoint than during unperturbed mitotic entry (Figure 4B, right panel). Taken together, our findings show that the balance between these two key mitotic inducers is changed during recovery from a DNA damage checkpoint.

## 4. Discussion

We have devised a method to sort cells based on kinase activity using a FRET-based reporter. As the method responds to a kinase/phosphatase balance, specific subsets of cells can be selected based on their signaling status. This allows selection of cells that have responded to a stimulus, but in which changes in protein content or morphology are minor or not yet apparent. Further, the enrichment of live cells can circumvent errors due to synchronization. The range of available FRET-based probes for kinase/phosphatase balances is rapidly increasing [28], and the last two decades have seen a rise in various cellular pathways studied by FRET-based reporters [29,30]. FRET-activated cell sorting (FRACS) could therefore be used to study a range of rare events.

By convenience, the cell cycle is divided into phases, although little evidence supports that cells in individual phases constitute a homogenous population. We use the setup to study G2 phase divided into populations based on the gradual accumulation of PLK1 activity. Our findings highlight the fact that protein content continuously changes through G2 phase and that cells in early G2 phase are distinct from cells in late G2 phase. During G2 phase, proteins that are required for cell division accumulate. In parallel to protein accumulation, we identify a set of proteins that decrease through G2 phase. The decrease could reflect that these proteins are not required after completion of S-phase, but could also indicate proteins that could interfere with mitotic progression, or that participate in signaling roles through G2 phase. We independently verify that ATAD2 is a protein that decreases through G2 phase and note that the decrease depends on proteasome activity. This emphasizes the presence of additional layers of regulation on top of the more studied transcriptional and translational changes that occur through the cell cycle [31,32,33]. Our findings agree with recent studies that uses FACS-based enrichment of fixed cells or large scale immunofluorescence of unsynchronized cells [34,35,36].

DNA damage enforces a block in cell cycle progression in G2 phase, at least in part by restricting the activity of pro-mitotic kinases. Spontaneous recovery from a cell cycle arrest in G2 phase is highly asynchronous, and typically concerns a small subset of cells at any time. We used the fact that PLK1 activity gradually increases through the recovery process to sort individual cells [24,25]. We note that Cyclin B1 levels continue to increase through a DNA damage arrest in G2 phase, with the consequence that the ratio between PLK1 activity and Cyclin B1 levels are altered once the cell cycle resumes. It is highly likely that the changed balance concerns more proteins and activities. The changed balance has two major consequences. First, mitotic cells that have recovered from a G2 checkpoint contain more Cyclin B1 than unperturbed mitotic cells. Mitotic progression is frequently disturbed after DNA damage, which is typically attributed to remaining DNA lesions in mitosis [37,38,39]. Our findings open up the possibility that mitotic progression is also affected by changed levels of mitotic regulators. Second, the changed balance between PLK1 activity and Cyclin B1 levels indicates that G2 phase is rewired after a DNA damage insult. Whereas complete inhibition of PLK1 is required to prevent unperturbed mitotic entry, partial inhibition is sufficient to block mitotic entry after recovery from a DNA damage response in G2 phase [13,40]. We observed that partial inhibition of CDK activity abrogated mitotic entry in unperturbed cells, whereas it only had a mild effect on mitotic entry after recovery from a DNA damage checkpoint in G2 phase. Our findings fit with a model in which both PLK1 and CDK1 are essential components of the signaling network that promotes mitotic entry, but, depending on the history of the cell, different parts of this network constitute the bottleneck for entering mitosis. The exact limitation of each kinase likely depends on the strength and duration of a DNA damage checkpoint, and finding exact values would require a careful titration for each condition. Our findings may have implications for the sensitivity to cell cycle kinase inhibitors after DNA-damaging cancer treatment.

## Figures and Tables

**Table 1:** Information on antibodies used in this article.

Antibody Target	Species	Supplier/Reference	Application
53BP1	Rabbit	Abcam, ab21083	IF
ATAD2	Rabbit	ATLAS antibodies HPA029424	IF, WB
Cyclin B1 (V152)	Mouse	Cell signaling #4135	IF, WB
Cyclin B1 (D-1)	Mouse	Santa Cruz, 166210	IF
pSer10-Histone H3 (D2C8)	Rabbit	Cell signaling #3377	IF, WB
pSer824-KAP1	Rabbit	Bethyl laboratories, A300-767A	WB
pSer/Thr-Pro MPM-2	Mouse	Merck Millipore, 05-368	IF
β-tubulin (93F)	Rabbit	Cell signaling, #2128	WB
pSer46-TCTP	Rabbit	Cell signaling, #5251	IF

## Supplementary Materials

The following are available online at https://www.mdpi.com/2073-4409/9/9/2126/s1, Figure S1: A setup to detect FRET by ratiometric flow cytometry, Figure S2: Characterization of ATAD2 levels through the cell cycle, Figure S3: A setup to investigate checkpoint recovery by FRACS, Figure S4: Quantification of pTCTP versus MPM-2 staining, supplementary data file: data from Mass Spectrometry analysis.

## References

[1] Bertoli C., Skotheim J.M., de Bruin R.A.M. (2013). Control of cell cycle transcription during G1 and S phases. Nat. Rev. Mol. Cell Biol..

[2] Stallaert W., Kedziora K.M., Chao H.X., Purvis J.E. (2019). Bistable switches as integrators and actuators during cell cycle progression. FEBS Lett..

[3] Lemmens B., Lindqvist A. (2019). DNA replication and mitotic entry: A brake model for cell cycle progression. J. Cell Biol..

[4] Fu Z., Malureanu L., Huang J., Wang W., Li H., van Deursen J.M., Tindall D.J., Chen J. (2008). Plk1-dependent phosphorylation of FoxM1 regulates a transcriptional programme required for mitotic progression. Nat. Cell Biol..

[5] Laoukili J., Alvarez M., Meijer L.A., Stahl M., Mohammed S., Kleij L., Heck A.J., Medema R.H. (2008). Activation of FoxM1 during G2 requires cyclin A/Cdk-dependent relief of autorepression by the FoxM1 N-terminal domain. Mol. Cell Biol..

[6] Marceau A.H., Brison C.M., Nerli S., Arsenault H.E., McShan A.C., Chen E., Lee H.-W., Benanti J.A., Sgourakis N.G., Rubin S.M. (2019). An order-to-disorder structural switch activates the FoxM1 transcription factor. Elife.

[7] Lemmens B., Hegarat N., Akopyan K., Sala-Gaston J., Bartek J., Hochegger H., Lindqvist A. (2018). DNA Replication Determines Timing of Mitosis by Restricting CDK1 and PLK1 Activation. Mol. Cell.

[8] Saldivar J.C., Hamperl S., Bocek M.J., Chung M., Bass T.E., Cisneros-Soberanis F., Samejima K., Xie L., Paulson J.R., Earnshaw W.C. (2018). An intrinsic S/G2 checkpoint enforced by ATR. Science.

[9] Li Z., Liu S., Yang Q. (2017). Incoherent Inputs Enhance the Robustness of Biological Oscillators. Cell Syst..

[10] Cooper S. (2019). The synchronization manifesto: A critique of whole-culture synchronization. FEBS J..

[11] Matson J.P., House A.M., Grant G.D., Wu H., Perez J., Cook J.G. (2019). Intrinsic checkpoint deficiency during cell cycle re-entry from quiescence. J. Cell Biol..

[12] Shaltiel I.A., Krenning L., Bruinsma W., Medema R.H. (2015). The same, only different - DNA damage checkpoints and their reversal throughout the cell cycle. J. Cell Sci..

[13] van Vugt M.A.T.M., Brás A., Medema R.H. (2004). Polo-like kinase-1 controls recovery from a G2 DNA damage-induced arrest in mammalian cells. Mol. Cell.

[14] Macurek L., Lindqvist A., Lim D., Lampson M.A., Klompmaker R., Freire R., Clouin C., Taylor S.S., Yaffe M.B., Medema R.H. (2008). Polo-like kinase-1 is activated by aurora A to promote checkpoint recovery. Nature.

[15] Peschiaroli A., Dorrello N.V., Guardavaccaro D., Venere M., Halazonetis T., Sherman N.E., Pagano M. (2006). SCFbetaTrCP-mediated degradation of Claspin regulates recovery from the DNA replication checkpoint response. Mol. Cell.

[16] Mamely I., van Vugt M.A., Smits V.A., Semple J.I., Lemmens B., Perrakis A., Medema R.H., Freire R. (2006). Polo-like kinase-1 controls proteasome-dependent degradation of Claspin during checkpoint recovery. Curr. Biol..

[17] Mailand N., Bekker-Jensen S., Bartek J., Lukas J. (2006). Destruction of Claspin by SCFbetaTrCP restrains Chk1 activation and facilitates recovery from genotoxic stress. Mol. Cell.

[18] van Vugt M.A.T.M., Gardino A.K., Linding R., Ostheimer G.J., Reinhardt H.C., Ong S.-E., Tan C.S., Miao H., Keezer S.M., Li J. (2010). A mitotic phosphorylation feedback network connects Cdk1, Plk1, 53BP1, and Chk2 to inactivate the G(2)/M DNA damage checkpoint. PLoS Biol..

[19] Lyutvinskiy Y., Yang H., Rutishauser D., Zubarev R.A. (2013). In silico instrumental response correction improves precision of label-free proteomics and accuracy of proteomics-based predictive models. Mol. Cell Proteomics.

[20] Schneider C.A., Rasband W.S., Eliceiri K.W. (2012). NIH Image to ImageJ: 25 years of image analysis. Nat. Methods.

[21] McQuin C., Goodman A., Chernyshev V., Kamentsky L., Cimini B.A., Karhohs K.W., Doan M., Ding L., Rafelski S.M., Thirstrup D. (2018). CellProfiler 3.0: Next-generation image processing for biology. PLoS Biol..

[22] Akopyan K., Lindqvist A., Mullers E. (2016). Cell Cycle Dynamics of Proteins and Post-translational Modifications Using Quantitative Immunofluorescence. Methods Mol. Biol..

[23] Akopyan K., Silva Cascales H., Hukasova E., Saurin A.T., Mullers E., Jaiswal H., Hollman D.A., Kops G.J., Medema R.H., Lindqvist A. (2014). Assessing kinetics from fixed cells reveals activation of the mitotic entry network at the S/G2 transition. Mol. Cell.

[24] Liang H., Esposito A., De S., Ber S., Collin P., Surana U., Venkitaraman A.R. (2014). Homeostatic control of polo-like kinase-1 engenders non-genetic heterogeneity in G2 checkpoint fidelity and timing. Nat. Commun..

[25] Jaiswal H., Benada J., Müllers E., Akopyan K., Burdova K., Koolmeister T., Helleday T., Medema R.H., Macurek L., Lindqvist A. (2017). ATM/Wip1 activities at chromatin control Plk1 re-activation to determine G2 checkpoint duration. EMBO J..

[26] Cucchi U., Gianellini L.M., De Ponti A., Sola F., Alzani R., Patton V., Pezzoni A., Troiani S., Saccardo M.B., Rizzi S. (2010). Phosphorylation of TCTP as a marker for polo-like kinase-1 activity in vivo. Anticancer Res..

[27] Yaffe M.B., Schutkowski M., Shen M., Zhou X.Z., Stukenberg P.T., Rahfeld J.U., Xu J., Kuang J., Kirschner M.W., Fischer G. (1997). Sequence-specific and phosphorylation-dependent proline isomerization: A potential mitotic regulatory mechanism. Science.

[28] Lin W., Mehta S., Zhang J. (2019). Genetically encoded fluorescent biosensors illuminate kinase signaling in cancer. J. Biol. Chem..

[29] Terai K., Imanishi A., Li C., Matsuda M. (2019). Two Decades of Genetically Encoded Biosensors Based on Förster Resonance Energy Transfer. Cell Struct. Funct..

[30] Zhang X., Hu Y., Yang X., Tang Y., Han S., Kang A., Deng H., Chi Y., Zhu D., Lu Y. (2019). FÖrster resonance energy transfer (FRET)-based biosensors for biological applications. Biosens. Bioelectron..

[31] Boström J., Sramkova Z., Salašová A., Johard H., Mahdessian D., Fedr R., Marks C., Medalová J., Souček K., Lundberg E. (2017). Comparative cell cycle transcriptomics reveals synchronization of developmental transcription factor networks in cancer cells. PLoS ONE.

[32] Giotti B., Chen S.-H., Barnett M.W., Regan T., Ly T., Wiemann S., Hume D.A., Freeman T.C. (2019). Assembly of a parts list of the human mitotic cell cycle machinery. J. Mol. Cell Biol..

[33] Stumpf C.R., Moreno M.V., Olshen A.B., Taylor B.S., Ruggero D. (2013). The translational landscape of the mammalian cell cycle. Mol. Cell.

[34] Ly T., Whigham A., Clarke R., Brenes-Murillo A.J., Estes B., Madhessian D., Lundberg E., Wadsworth P., Lamond A.I. (2017). Proteomic analysis of cell cycle progression in asynchronous cultures, including mitotic subphases, using PRIMMUS. Elife.

[35] Kelly V., al-Rawi A., Lewis D., Ly T. (2020). Cell cycle state proteomics and classification using in-cell protease digests and mass spectrometry. bioRxiv.

[36] Mahdessian D., Cesnik A.J., Gnann C., Danielsson F., Stenström L., Arif M., Zhang C., Shutten R., Bäckström A., Thul P. (2020). Spatiotemporal dissection of the cell cycle with single-cell proteogenomics. bioRxiv.

[37] Chan Y.W., Fugger K., West S.C. (2018). Unresolved recombination intermediates lead to ultra-fine anaphase bridges, chromosome breaks and aberrations. Nat. Cell Biol..

[38] Mikhailov A., Cole R.W., Rieder C.L. (2002). DNA damage during mitosis in human cells delays the metaphase/anaphase transition via the spindle-assembly checkpoint. Curr. Biol..

[39] Deckbar D., Birraux J., Krempler A., Tchouandong L., Beucher A., Walker S., Stiff T., Jeggo P., Löbrich M. (2007). Chromosome breakage after G2 checkpoint release. J. Cell Biol..

[40] Gheghiani L., Loew D., Lombard B., Mansfeld J., Gavet O. (2017). PLK1 Activation in Late G2 Sets Up Commitment to Mitosis. Cell. Rep..

