# Supplementary material for: FRET-Based Sorting of Live Cells Reveals Shifted Balance between PLK1 and CDK1 Activities During Checkpoint Recovery
Source: Cells. 2020 Sep 19;9(9):2126. doi: 10.3390/cells9092126 (PMC7564076; doi:10.3390/cells9092126)
Supplement: Supplementary file 1 [file cells-09-02126-s001.zip › cell-923790-supplementary/supplementary figures.pdf]

cells

MDPI

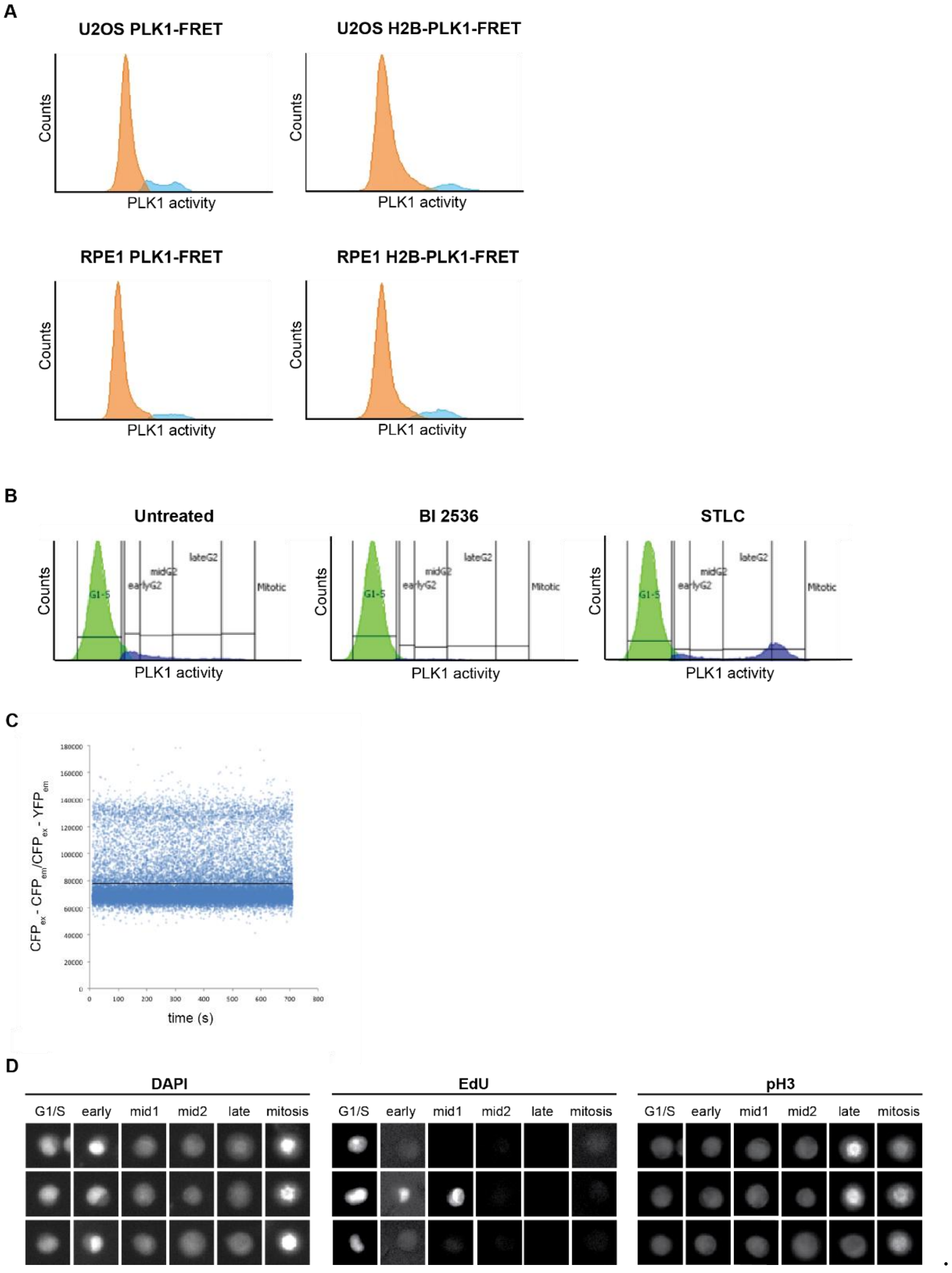

Figure S1. A setup to detect FRET by ratiometric flow cytometry. (**A**) Populations of asynchronously-growing U2OS and RPE1 cells expressing either a diffusible PLK1-FRET probe or a Histone H2B-coupled PLK1-FRET probe were visualized by flow cytometry. (**B**) RPE1 cells were either left untreated (left panel), treated for 2 hours with the PLK1 inhibitor BI 2536 (middle panel) or treated for 6 hours with STLC (right panel) and then visualized by flow cytometry. (**C**) PLK1 activity of a population of RPE1 cells resuspended in ice-cold PBS was measured by flow cytometry over 30 minutes. The conditions used throughout the study allow a stable readout for a prolonged period of time. (**D**) Representative pictures of cells quantified in Fig 1D. After being sorted based on PLK1 activity, RPE1 cells were stained for EdU, phospho-Histone H3 and DAPI.

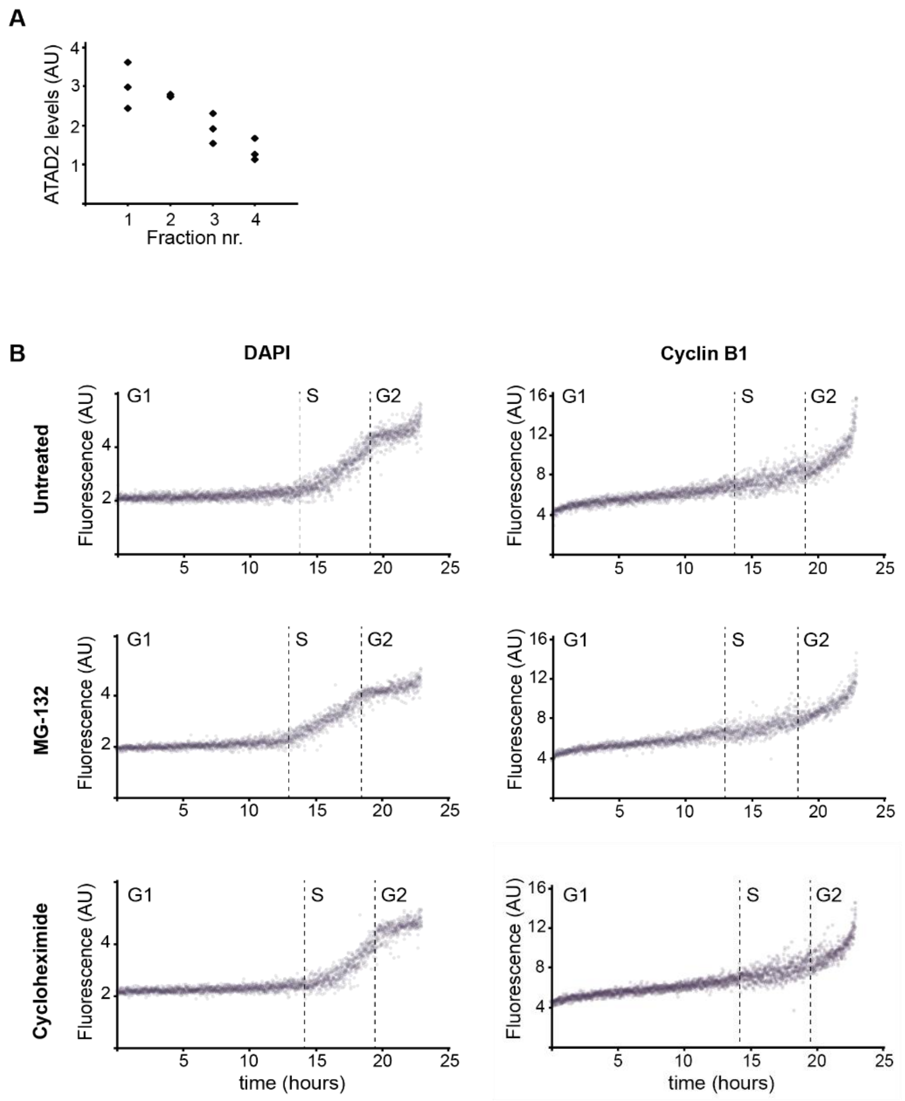

Figure S2. Characterization of ATAD2 levels through the cell cycle. (**A**) Mass spectrometric analysis of cells sorted according to their levels of PLK1 activity revels that ATAD2 levels decrease during G2 phase. (**B**) Quantitative immunofluorescence of DAPI and cyclin B1 levels throughout the cell cycle. Two hours prior to fixation asynchronously growing RPE1 cells were treated with either DMSO, 10 μM MG-132 or 10 μg/ml cycloheximide (CHX). Samples were stained with antibodies against Cyclin B1 and ATAD2, and counterstained with DAPI. Following image acquisition and quantification, cells were sorted *in silico* and assigned to their position within the cell cycle based on their DAPI and Cyclin B1 intensity levels. Every dot on the plot represents a single cell and the trend line is the running average of 30 cells.

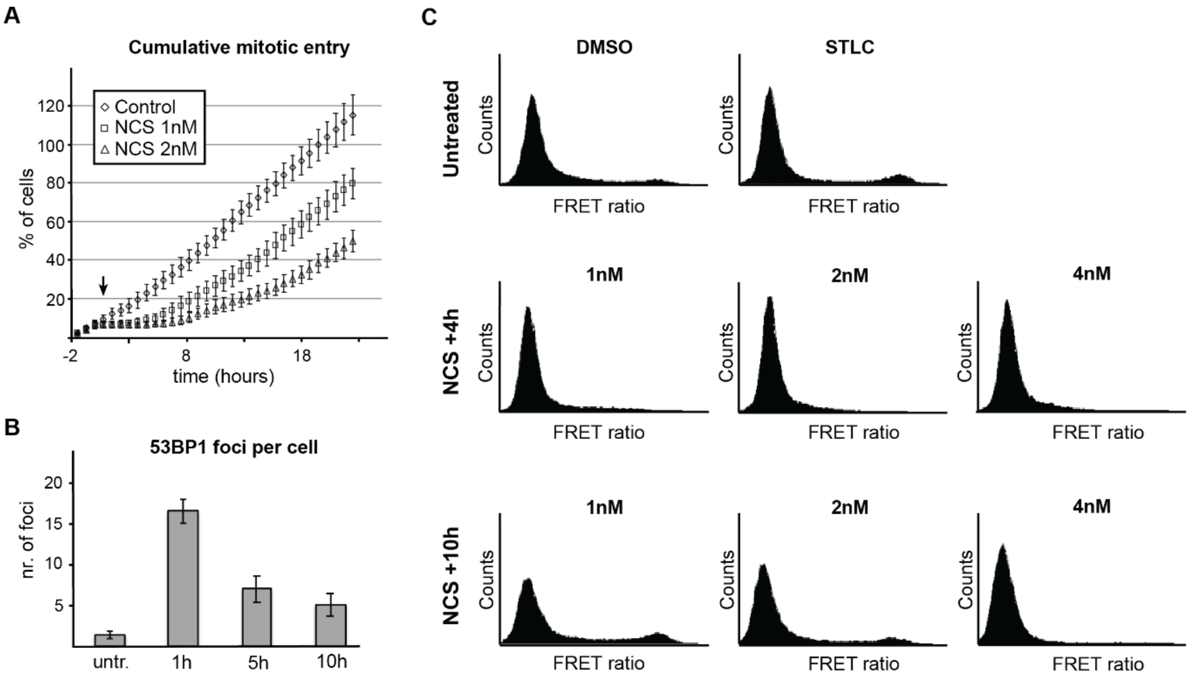

Figure 3. A setup to investigate checkpoint recovery by FRACS. (**A**) Cumulative mitotic entry of U2OS cells treated with different concentrations of NCS was recorded by live cell imaging. The data are reported as mean of four independent experiments and the error bars show the standard error of the mean. (**B**) Repair kinetics of double strand breaks (DSBs) in U2OS cells is analyzed by quantifying the number of 53BP1 foci per cells at different time-points after treatment with 2 nM NCS. The graph presents the mean and standards error of three independent experiments. (**C**) U2OS cells were treated with different concentration of NCS. Living cells were analyzed by flow cytometry at 4 or 10 hours after damage induction to assess the levels of PLK1 activity. Four hours before the analysis, cells were treated with 10 μM S-trityl-L-cysteine (STLC) to prevent mitotic cells to re-enter the cell cycle.

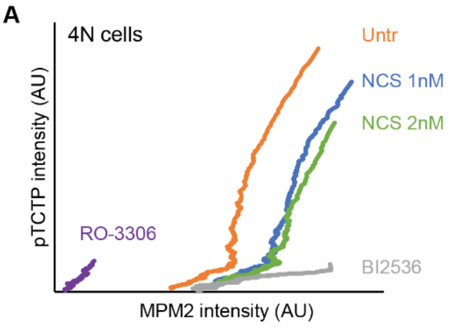

Figure S4. Quantification of pTCTP versus MPM-2 staining. (**A**) Running median of pTCTP versus MPM-2 cellular levels of all the G2 cells identified in the experiment shown in (A). Included are control cells treated with either 24 μM of the CDK1 inhibitor RO-3306 or 20 nM of the PLK1 inhibitor BI 2536. The high concentration of RO-3306 and BI 2536 were applied to ensure a high level of inhibition of CDK1 and PLK1 to define a base-line of phosphorylation-dependent staining for pTCTP and MPM-2 antibodies.
